# A new method to quantify left ventricular mass by 2D echocardiography

**DOI:** 10.1038/s41598-022-13677-1

**Published:** 2022-06-15

**Authors:** Charlotte Burup Kristensen, Katrine Aagaard Myhr, Frederik Fasth Grund, Niels Vejlstrup, Christian Hassager, Raj Mattu, Rasmus Mogelvang

**Affiliations:** 1 Department of Cardiology, The Heart Center, Rigshospitalet – University hospital of Copenhagen, Blegdamsvej 9, 2100 Copenhagen, Denmark; 2 Institute of Clinical Medicine, Faculty of Health and Medical Sciences, University of Copenhagen, Blegdamsvej 3B, 2100 Copenhagen, Denmark; 3 Kettering General Hospital NHS Foundation Trust, Kettering, NN16 8UZ Northants UK; 4 University College London, Gower St, London, WC1E 6BT UK; 5 Cardiovascular Research Unit, University of Southern Denmark, Baagoees allé 15, 5700 Svendborg, Denmark

**Keywords:** Cardiology, Translational research

## Abstract

Increased left ventricular mass (LVM) is a strong independent predictor for adverse cardiovascular events, but conventional echocardiographic methods are limited by poor reproducibility and accuracy. We developed a novel method based on adding the mean wall thickness from the parasternal short axis view, to the left ventricular end-diastolic volume acquired using the biplane model of discs. The participants (n = 85) had various left ventricular geometries and were assessed using echocardiography followed immediately by cardiac magnetic resonance, as reference. We compared our novel two-dimensional (2D) method to various conventional one-dimensional (1D) and other 2D methods as well as the three-dimensional (3D) method. Our novel method had better reproducibility in intra-examiner [coefficients of variation (CV) 9% vs. 11–14%] and inter-examiner analysis (CV 9% vs. 10–20%). Accuracy was similar to the 3D method (mean difference ± 95% limits of agreement, CV): Novel: 2 ± 50 g, 15% vs. 3D: 2 ± 51 g, 16%; and better than the “linear” 1D method by Devereux (7 ± 76 g, 23%). Our novel method is simple, has considerable better reproducibility and accuracy than conventional “linear” 1D methods, and similar accuracy as the 3D-method. As the biplane model forms part of the standard echocardiographic protocol, it does not require specific training and provides a supplement to the modern echocardiographic report.

## Introduction

Increased left ventricular mass (LVM) is a strong independent predictor for adverse cardiovascular events^1–3^, and associated with impaired left ventricular (LV) myocardial function, coronary artery disease and arrhythmogenesis^4^. Unfavourable associations with increased LVM seem reversible through reduction of LVM^5^, but clinical responses to treatment and prognosis using echocardiography requires reliable LVM-quantification. As conventional methods for LVM-quantification lack reproducibility they are not suitable for serial comparisons, thereby not routinely deployed on individuals. This warrants a method with greater reproducibility to improve accuracy in detecting actual differences. Standard one-dimensional linear echocardiography (1DE) for LVM-quantification by the cube formula relies on a symmetrical shaped left ventricle (LV). Whilst technically simple, it is prone to inaccuracies^6^ and more suitable for comparison on a population level. Conversely, three-dimensional echocardiography (3DE) is independent of LV symmetry and has higher concordance with the reference method cardiac magnetic resonance (CMR)^7,8^. Since acquisition and analysis using 3DE are challenging and time-consuming, this presents disadvantages in busy echocardiography labs. We explored an alternative method to preserve the geometrical shape of the LV by applying the biplane model of discs by two-dimensional echocardiography (2DE) for LVM-quantification, without the need for troublesome epicardial boundary delineation in the apical views.

Our aims were to:

1. Develop a simpler, feasible and reproducible 2DE-based method for LVM-quantification that is less dependent on LV symmetry.
2. Compare various well-known echocardiographic methods for LVM-quantification as well as our novel method to CMR, amongst subjects with assorted LV geometries.

## Methods

### Study design

This is a single-centre prospective cohort feasibility study. We included patients scheduled for echocardiography > 18 years with sinus rhythm on the study days. Pregnant, breastfeeding, or claustrophobic patients were excluded. We aimed to include a wide variety of subjects with different LV geometries. All subjects were assessed at “baseline” using echocardiography immediately followed by CMR and re-assessed at “re-examination” using echocardiography after a median of 6 days (IQR 3–18). To limit effects of hydration status, all subjects were instructed to abstain from oral intake ≥ 5 h prior to both visits. The study was conducted in accordance with the second Helsinki declaration and approved by the Clinical Research Unit of the Heart Centre at the Rigshospitalet University Hospital of Copenhagen and by the local ethics committee of the Capital Region of Denmark, Protocol number H-16029778. All participants provided written informed consent.

### Echocardiography: acquisition and analysis

**Figure 1:**
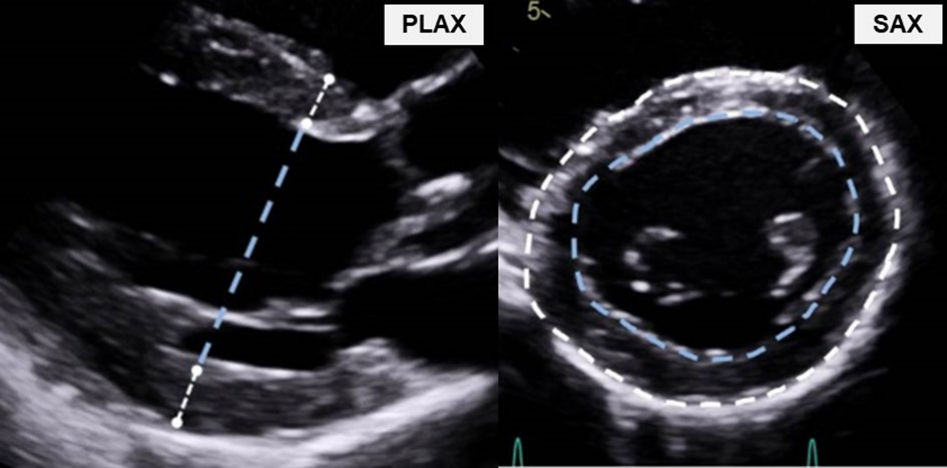
Wall thickness measurements at the chordae level. *PLAX* parasternal long-axis, *SAX* parasternal short-axis.

One experienced sonographer performed all examinations at both baseline and re-examination, using a Vivid E95 ultrasound scanner (GE Healthcare, Norway), and M55c-D-matrix-array transducer (1.5–4.6 MHz) for 2DE and a 4 V-D volume-phased array transducer (1.5–4 MHz) for 3DE. Subjects were studied in the left lateral decubitus position with parasternal long-axis view (PLAX), short-axis view (SAX), apical four-chamber view (4CH), apical two-chamber view (2CH), and 3DE. We reduced depth to focus on the LV. Framerate for 2DE was 65 ± 7 frames/s, and for 3DE 26 ± 8 volumes/s. The 3DE full volume dataset was acquired from the apical window during breath-hold over four to six heart beats. The examinations were analysed using EchoPAC version 201.61 (GE Healthcare, Norway). End-diastole was defined as the first frame of mitral valve closure. We distinguished between the end-diastolic-volume (EDV) defined by the inner myocardial interface [endocardium (EDV_ENDO_)] and by the outer myocardial interface [epicardium (EDV_EPI_)]. Conventional EDV (EDV_ENDO_) was quantified by 3DE, 2DE using the biplane model^9^ and 1DE using the Teichholtz model^10^. LVM-quantifications were made at end-diastole. ECG-timing from PLAX was referenced to find the corresponding SAX-frame. All PLAX/SAX-measurements were made at the chordae level (Fig. 1). In PLAX the region between the mitral valve and papillary muscle, just beneath the attachment of the chordae to the papillary muscle. In SAX this corresponded to the visible attachments of the chordae to the papillary muscle. In this view, the mitral valve should not be visible, and chordae should be separated from LV wall. We delineated the boundaries in SAX by using the blood-endocardium interface, the inner boundary delineation and by the epicardium-blood/pericardium interface, outer boundary delineation. We didn’t use a leading-to-leading edge approach. Trabeculae or papillary muscles were considered part of the LV cavity, the pericardium was excluded from the delineation. 3DE LVM was quantified by the vendor-specific software package 4D Auto LVQ (EchoPAC, GE Healthcare, Norway). The full volume dataset was aligned for three apical views, which were manually adjusted, guided by the derived short-axis views.

### The novel method

**Figure 2:**
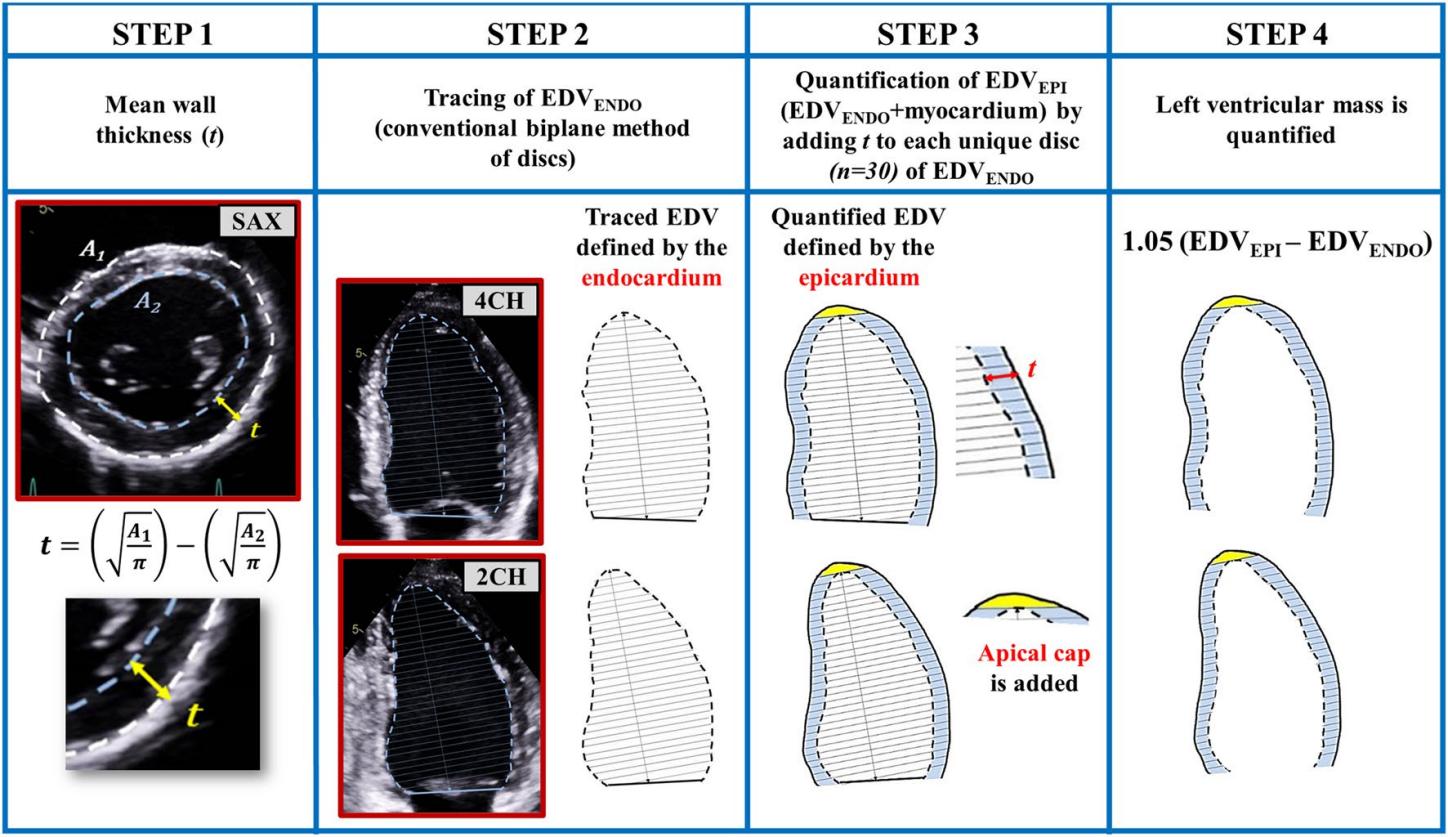
The novel method. *Step 1* Mean wall thickness (*t*) is calculated by delineation of the endocardial and epicardial border in the parasternal short axis view at the chordae level. *Step 2* Conventional delineation in the apical four- and two-chamber view and for end-diastolic volume according to the biplane model of discs. *Step 3* The total volume defined by the epicardium is quantified by adding *t* to each unique disc from the delineations in step 2. An apical cap with the geometrical assumption of a prolate ellipsoid is added. *Step 4* Left ventricular myocardial volume is quantified by subtracting the traced volume defined by the endocardium (from step 2) from the quantified volume defined by the epicardium (from step 3). Left ventricular mass is quantified my multiplying the left ventricular volume with the myocardial density/gravity of 1.05 g/ml. *t *mean wall thickness, *A*_1_ outer (epicardial) delineation, *A*_2_ inner (endocardial) delineation, SAX short-axis view, EDV end-diastolic volume, 4CH four-chamber view, 2CH two-chamber view.

The novel method of LVM-quantification is based on adding the mean wall thickness (*t*) from a single SAX-recording to the EDV_ENDO_ acquired by the standard biplane model of discs in the apical 4CH- and 2CH-view. The *t* is calculated from SAX by conversion from the traced myocardial area, in the same manner as for the conventional 2DE methods (Step 1, Fig. 2). The EDV_ENDO_ is the sum of the sub-volumes of 30 unique discs, which are acquired during the conventional biplane delineation in the apical views. (Step 2, Fig. 2) A factor (*k*) adjusts the echocardiographic EDV_ENDO_ with CMR derived EDV_ENDO_. EDV_EPI_ is the summation of 30 larger sub-volumes quantified by adding *t* to each unique sub-volume from the EDV_ENDO_-delineation. An apical cap is added for EDV_EPI_. (Step 3, Fig. 2) The difference between the quantified EDV_EPI_ and the traced EDV_ENDO_ was multiplied with the myocardial gravity of 1.05 g/ml. (Step 4, Fig. 2). EDV_ENDO_-delineations from each apical view including information regarding size of each unique disc from was exported from EchoPac and quantification of LVM was performed in Windows Excel 2010 (Microsoft Office Professional Plus). A more detailed description of the novel method, including formula for software implementation is available in the supplementary data.

### Left ventricular mass quantification using echocardiography

**Figure 3:**
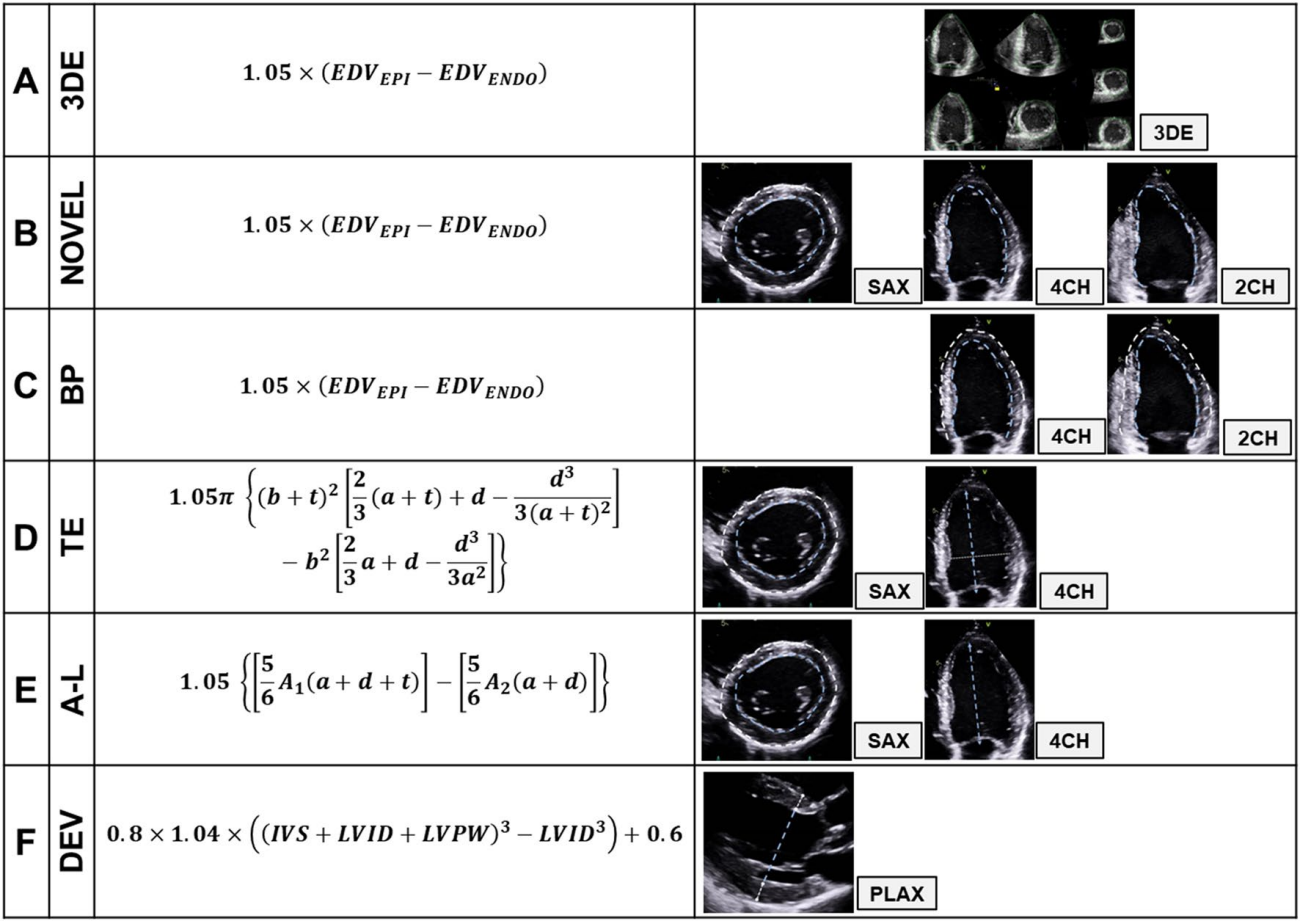
(**A–F**) Various methods for quantification of left ventricular mass. *3DE* three-dimensional echocardiography, *EDV*_*EPI*_ end-diastolic volume defined by the epicadium, *EDV*_*ENDO*_ end-diastolic volume defined by the endocardium, *NOVEL* novel method, *t* mean wall thickness, *SAX* short-axis, *4CH* four-chamber, *2CH* two-chamber, *BP* biplane model (both endo- and epicardial delineation), *TE* truncated ellipsoid, *a* length, apex to short-axis-plane, *d* length, short-axis-plane to mitral-plane, *A-L *area-length, *A*_*2*_ inner (endocardial) area (blue dotted lines) short-axis, *A*_*1*_ outer (epicardial) area (white dotted lines) short-axis, *DEV* cube formula, Devereux correction, *IVS* interventricular septum, *LVID* left ventricular internal diameter, *LVPW* left ventricular posterior wall. $b = \left( {\sqrt {\frac{{A_{2} }}{\pi }} } \right)$ $t = \left( {\sqrt {\frac{{A_{1} }}{\pi }} } \right) - \left( {\sqrt {\frac{{A_{2} }}{\pi }} } \right)$.

We evaluated six different methods for LVM-quantification (Fig. 3)^11–14^. Four of these are widely recognized; *A*, *D*, *E*, *F*. All except *B* the novel method and *C* endo- and epicardial delineation in the 4CH- and 2CH-view by the biplane model of discs (BP), are recommended in current guidelines^6^.

### Cardiac magnetic resonance: acquisition and analysis

Accuracy of the echocardiographic methods was defined by agreement according to CMR^15–17^. CMR images were obtained using a 1.5 Tesla system (GE Optima MR450W, GE Healthcare, Waukesha, WI) with a phased-array cardiac coil. Cine images were acquired during breath-hold using a steady-state free precession cine sequence with retrospective gating^18^. Slice thickness 8 mm, no gaps, field of view 300–360 mm, 25 phases/cycle. Analysis was performed using CVI42 (Circle Cardiovascular Imaging Inc., Version 5.6.5, Canada). End-diastole was defined as for echocardiography. Endocardium and epicardium were manually delineated in the short-axis-stack, papillary muscles were considered part of the LV volume. The subjects were classified in four groups according to age, gender and indexed values^19^ of the EDV_ENDO_ and LVM^20^; normal, dilatation, hypertrophy, dilatation and hypertrophy.

### Reproducibility

Intra/inter-examiner examination were compared at baseline and test–retest variation between baseline and re-examination. All subjects were asked to walk around between baseline examinations and intra/inter-examiner exam. Intra-examiner exams (n = 13) were acquired and analysed by the same examiner who performed the baseline and re-examination exams, inter-examiner exams (n = 20) were acquired and analysed by another examiner.

### Feasibility

Feasibility was estimated for the entire study cohort and for a small ‘all-comers’ cohort of twenty-six consecutive patients examined by a third sonographer during one week in our echo lab, no patients were excluded. Since 3DE is not routinely performed on all patients at our echo lab, we are unable to report reliable “all-comers” 3DE feasibility.

### Classification of hypertrophy versus non-hypertrophy

Hypertrophy was defined by CMR according to age, gender and LVM-index^20^. The normal LVM-ranges for CMR were applied for the echocardiographic 3DE, novel method and BP-method^20^. Current echocardiographic guidelines were applied for normal LVM-ranges for the cube formula by Devereux (DEV), truncated ellipsoid (TE) and area-length (A-L)^6^. Sensitivity, specificity, positive predictive value (PPV) and negative predictive value (NPV) for detection of hypertrophy was evaluated.

### Image quality

Image quality was graded as optimal, “analysis without effort” or suboptimal, “analysis with effort” or inadequate, “adequate analysis not possible”. We evaluated the image quality for each view separately; PLAX, SAX, apical views, 3D. For each method we evaluated the impact of image quality on the agreement to CMR and the test–retest variability.

### Statistics

Continuous variables were expressed as mean and standard deviation (SD) and categorical values expressed as frequencies and percentage. The accuracy of echocardiographic methods was defined according to agreement with CMR; evaluated by the Bland–Altman-method (BA) using paired t-test presented as mean difference (bias), 95% limits of agreement (LOA)^21^, simple linear regression, Pearson’s correlation and intra class correlation coefficient (ICC). Reproducibility was assessed by the 95%LOA and by the coefficient of variation (CV) presented as percentages. P-values < 0.05 were considered statistically significant. Data analysis was performed in SPSS v25.0 (IBM Corp. IBM SPSS Statistics for Windows, Version 25.0. Armonk, NY).

## Results

### Study population

All 85 subjects had echocardiography and CMR at baseline. All were re-invited for re-examination; four subjects cancelled in advance, one subject was sent home because of technical problems, one subject did not show up for re-examination. Baseline characteristics of the population are presented in Table 1. Cardiac condition according to LV geometry in supplementary data, Table S5. Baseline EDV_ENDO_ and LVM by various methods in Table 2. We included all data for every methodology, although some subjects did not have feasible images for all methods. Data on the subjects with 100% feasibility (n = 59) are specified in supplementary data Table S7.

**Table 1:** Baseline characteristics (n = 85).

Age (years)	44 ± 14
Male gender	57(67%)
Body mass index (kg/m^2^)	25.5 ± 4.2
Body surface area (m^2^)	2.0 ± 0.2
Systolic blood pressure (mmHg)	127 ± 18
Diastolic blood pressure (mmHg)	76 ± 13
Heart rate (bpm)	57 ± 8
**Cardiac disease**	41 (48%)
HCM	16 (19%)
DCM	2 (2%)
ARVC	1 (1%)
Moderate-severe aortic valve stenosis	6 (7%)
Moderate-severe aortic valve regurgitation	6 (7%)
IHD	3 (4%)
Others	7 (8%)
**Cardiovascular risk factors**	44 (52%)
Hypertension	17 (20%)
Diabetes	3 (4%)
Current/previous smoker	37 (44%)
Peripheral artery disease	1 (1%)
Stroke/TIA	2 (2%)
Physical inactivity	8 (9%)

*HCM* hypertrophic cardiomyopathy, *DCM* dilated cardiomyopathy, *ARVC* arrythmogenic right ventricular cardiomyopathy, *IHD* ischemic heart disease, *TIA* transient ischemic attack.

**Table 2:** End-diastolic volumes and left ventricular mass, baseline (n = 85).

	Mean ± SD
End-diastolic volume (ml)*	
CMR	197 ± 60
3DE	147 ± 51
2DE (BP)	151 ± 50
1DE (Teichholtz)	131 ± 43
**Left ventricular mass (g)**	
CMR	165 ± 62
3DE	168 ± 56
2DE (NOVEL)	167 ± 62
2DE (BP)	178 ± 66
2DE (TE)	163 ± 60
2DE (A-L)	187 ± 68
1DE (DEV)	172 ± 70

*Delineated by the endocardium, EDV_ENDO_.

*CMR* cardiac magnetic resonance, *3DE* three-dimensional echocardiography, *2DE* two-dimensional echocardiography, *1DE* one-dimensional echocardiography, *BP* biplane model (endocardial delineation for end-diastolic volume, endo- and epicardial delineation for left ventricular mass), *TE* truncated ellipsoid**,** *A-L* area-length, *DEV* cube formula, Devereux.

### Feasibility and reproducibility

We report high feasibility for all methods, except the BP-method (74% vs. 95–100%) (Table 3). All-comers’ feasibility was lower; DEV 92%, TE/A-L/Novel 81%, BP 50%. We observe similar test–retest-variations of the 2D/3D-methods (14–18%) and larger test–retest-variation of the 1DE-method DEV (21%) (Table 3). The novel method has better reproducibility in intra- (CV 9% vs. 11–14%) and inter-examiner (CV 9% vs. 10–20%) analysis (Table 3).

**Table 3:** Feasibility and Reproducibility.

	Feasibility (%)	Test–retest-variation			Intra-examiner-variation			Inter-examiner-variation		
		Bias ± 95%LOA	CV (%)	ICC	Bias ± 95%LOA	CV (%)	ICC	Bias ± 95%LOA	CV (%)	ICC
3DE	98	4 ± 45	*14*	0.96	− 8 ± 51	*14*	0.94	6 ± 54	*17*	0.77
NOVEL	95	− 4 ± 48	*15*	0.95	− 1 ± 27	*9*	0.98	4 ± 27	*9*	0.96
BP	74	− 3 ± 63	*18*	0.94	− 3 ± 47	*14*	0.92	9 ± 45	*15*	0.86
TE	95	− 5 ± 53	*15*	0.94	− 3 ± 34	*11*	0.97	0 ± 30	*10*	0.96
A-L	95	− 4 ± 47	*15*	0.95	− 1 ± 40	*11*	0.97	2 ± 34	*10*	0.96
DEV	100	− 1 ± 71	*21*	0.92	− 21 ± 45*	*13*	0.93	14 ± 58	*20*	0.86

*p < 0.05.

Test–retest-variation (n = 79), intra-examiner (n = 13) and inter-examiner (n = 20) analysis.

Test–retest variation is the comparison between two different days with the same examiner and same reader.

*LOA* limits of agreement, *CV* coefficient of variation, *3DE* three-dimensional echocardiography, *BP* biplane model (both endo- and epicardial delineation), *TE* truncated ellipsoid, *A-L* area-length, *DEV* cube, Devereux.

### Agreement of LVM quantification by echocardiography and CMR

**Figure 4:**
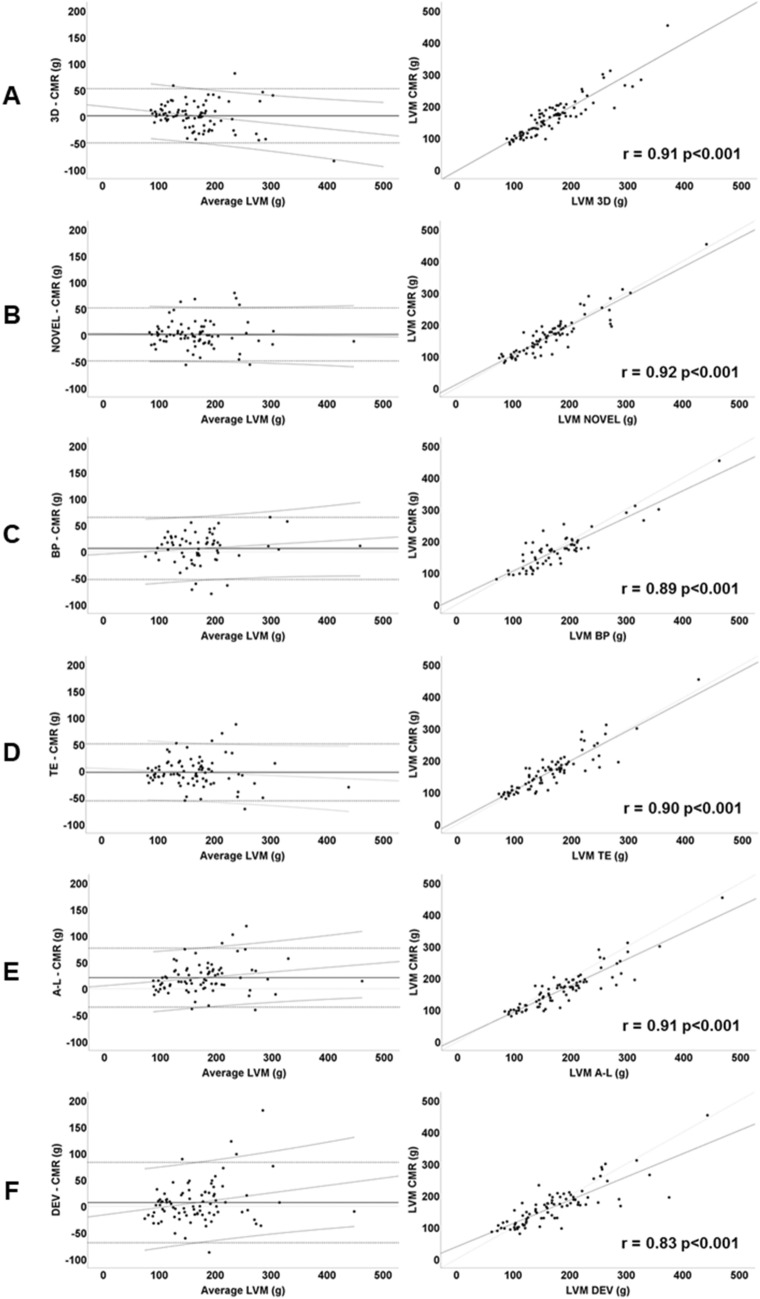
Agreement of left ventricular mass by echocardiography and cardiac magnetic resonance at baseline. *Left* Bland–Altman plots. Horizontal solid line = bias (mean difference). Horizontal dashed line = 95% limits of agreement. Solid/dashed diagonal lines = regression lines with 95% confidence interval. *Right* Linear regression, pearson’s correlation. (**A**) 3DE (**B**) Novel (**C**) Biplane model (**D**) Truncated ellipsoid (**E**) Area-length (**F**) Cube formula, Devereux correction. *LVM* left ventricular mass, *CMR* cardiac magnetic resonance.

**Figure 5:**
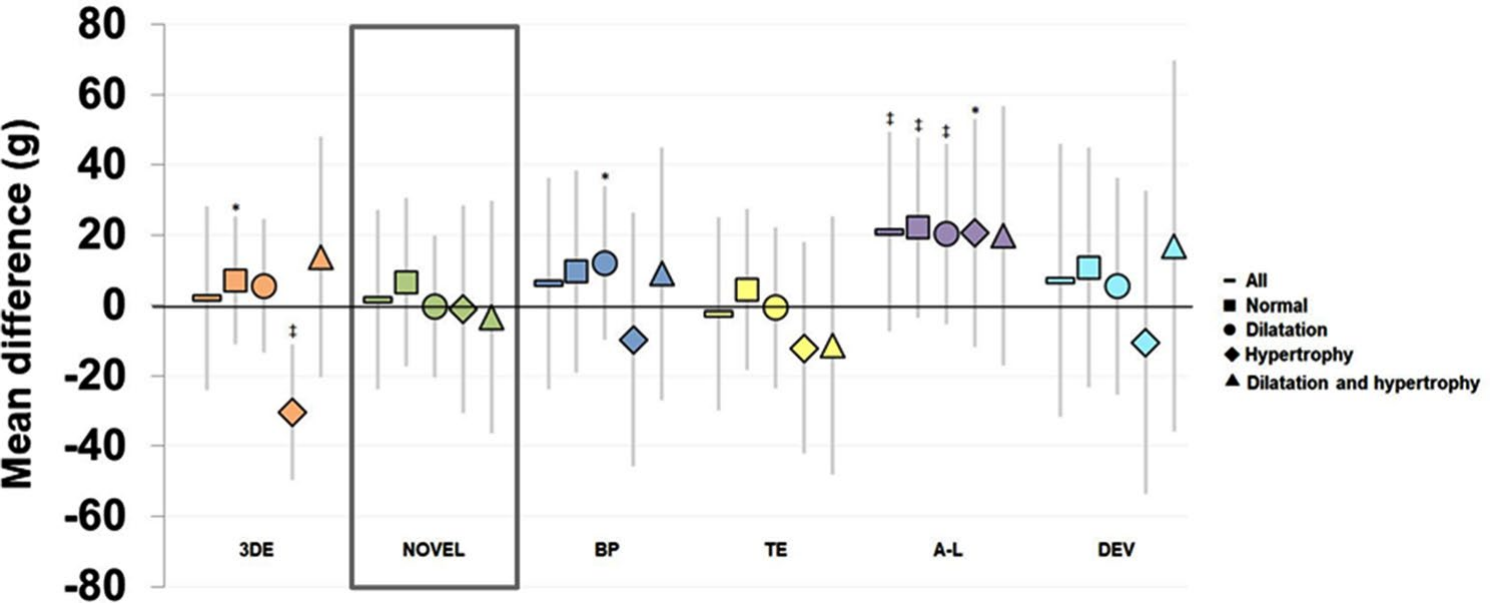
Agreement of left ventricular mass by echocardiography and cardiac magnetic resonance at baseline divided by geometry. Mean differences (g) between echocardiography and cardiac magnetic resonance; positive value indicates overestimation by echocardiography. Longitudinal grey solid line is standard deviation. *p < 0.0, ^†^p < 0.01 ^‡^p < 0.001. Definition of left ventricular geometry by cardiac magnetic resonance; normal, dilatation, hypertrophy, dilatation and hypertrophy. Dilatation defined as the following indexed end-diastolic volume values^20^: Men < 60 years ≥ 101 ml/m^2^, Men ≥ 60 years ≥ 95 ml/m^2^, Women < 60 years ≥ 96 ml/m^2^, Women ≥ 60 years 87 ml/m^2^. Hypertrophy defined as the following indexed left ventricular mass values^20^: Men < 60 years ≥ 92 g/m^2^, Men ≥ 60 years ≥ 91 g/m^2^, Women < 60 years ≥ 78 g/m^2^, Women ≥ 60 years 79 g/m^2^. *3DE* three-dimensional echocardiography, *BP* biplane model (both endo- and epicardial delineation), *TE* Truncated Ellipsoid, *A-L* Area-Length, *DEV* cube formula, Devereux correction.

Baseline agreements between echocardiography and CMR are visualized in BA-plots and linear regression-plots (Fig. 4). The novel method demonstrates equal distribution and limited proportional bias, based on the regression line (Fig. 4B). Table 4 presents the agreements between echocardiography and CMR at baseline. Figure 5 demonstrates the agreement of echocardiography and CMR among the defined LV geometries.

**Table 4:** Agreement between left ventricular mass by echocardiography and cardiac magnetic resonance, baseline.

	Baseline (*n* = 85)	
	Bias ± 95%LOA	CV (%)
3DE	2 ± 51	*16*
NOVEL	2 ± 50	*15*
BP	6 ± 59	*17*
TE	− 2 ± 54	*17*
A-L	21* ± 56	*16*
DEV	7 ± 76	*23*

*p < 0.05.

*LOA* limits of agreement, *CV* coefficient of variation, *3DE* three-dimensional echocardiography**,** *BP* biplane model (both endo- and epicardial delineation), *TE* truncated ellipsoid, *A-L* area-length, *DEV* cube, Devereux.

### Impact of image quality

Table 5 presents the agreement between LVM from echocardiography compared to CMR and the test–retest variability with comparison between exams performed on different days. The novel method is equally affected by suboptimal image quality as the other methods.

**Table 5:** The impact of image quality on echocardiographic evaluation of left ventricular mass.

Method	View	Optimal				Suboptimal			
		Agreement to cardiac magnetic resonance		Test–retest variability		Agreement to cardiac magnetic resonance		Test–retest variability	
		Bias ± 95%LOA	CV (%)	Bias ± 95%LOA	CV (%)	Bias ± 95%LOA	CV (%)	Bias ± 95%LOA	CV (%)
3DE	*3D*	1 ± 46	*14*	6 ± 43	*12*	3 ± 59	*19*	1 ± 49	*16*
NOVEL	*SAX* + *Apical*	− 5 ± 41	*12*	0 ± 46	*14*	9 ± 55	*17*	− 8 ± 48	*16*
BP	*Apical*	4 ± 62	*18*	0 ± 60	*17*	13 ± 49	*15*	− 13 ± 71	*19*
TE	*SAX* + *Apical*	− 11 ± 46	*14*	0 ± 45	*14*	6 ± 56	*18*	− 9 ± 48	*16*
A-L	*SAX* + *Apical*	13 ± 43	*12*	0 ± 51	*14*	29 ± 63	*19*	− 10 ± 53	*16*
DEV	*PLAX*	4 ± 71	*22*	3 ± 64	*20*	23 ± 97	*24*	− 33 ± 92	*22*

*Optimal image quality* = analysis without effort. *Suboptimal image quality* = analysis with effort.

Each method is evaluated according to the echocardiographic image quality of the represented view for each method. Test–retest variability was evaluated comparing echocardiographic exams from two different days.

*3DE* three-dimensional echocardiography, *BP* biplane model (both endo- and epicardial delineation), *TE* truncated ellipsoid, *A-L* area-length, *DEV* cube formula Devereux correction, *LOA* limits of agreement, *CV* coefficient of variation.

### Diagnostic performance on detecting hypertrophy using echocardiography

The sensitivity, specificity, PPV and NPV for all methods and divided by LV geometry are presented in Table 6.

**Table 6:** Diagnostic performance on detecting hypertrophy.

	Sensitivity (%)	Specificity (%)	PPV (%)	NPV (%)
3DE	71	87	74	86
NOVEL	82	95	88	92
BP	82	73	62	88
TE	52	98	93	80
A-L	85	89	79	92
DEV	36	96	83	75

*3DE* three-dimensional echocardiography, *BP* biplane model (both endo- and epicardial delineation), *TE* truncated ellipsoid, *A-L* area-length, *DEV* cube, Devereux , *PPV* positive predictive value, *NPV* negative predictive value.

Definition of hypertrophy:

3DE, NOVEL, BP^20^: Men < 60 years ≥ 92 g/m^2^, Men ≥ 60 years ≥ 91 g/m^2^, Women < 60 years ≥ 78 g/m^2^, Women ≥ 79 g/m^2^.

TE, A-L^6^: Men ≥ 103 g/m^2^, Women ≥ 89 g/m^2^.

DEV^6^: Men ≥ 116 g/m^2^, Women ≥ 96 g/m^2^.

### Suggested normal reference interval for the novel method

Twenty-six subjects (31%) had no cardiac disease and no cardiovascular risk factors. The 95% CI for this subgroup was 46–96 g/m^2^; women 39–91 g/m^2^, men 63–94 g/m^2^.

## Discussion

The main findings of our study are;

1. Our novel method has high feasibility and better intra/inter-examiner reproducibility than the other methods, especially the conventional “linear” 1DE-methods.
2. Accuracy of our novel method is similar to 3DE and greater across all four defined LV geometries than the other methods.
3. Our novel method is simple, does not require specific training and should not cause any considerable delay, since all images already form part of the standard echocardiographic protocol.
4. The formula can easily be integrated in any vendor specific echocardiographic analysis software for fast automatic quantification, and has the potential to provide a useful supplement to the modern echocardiographic report.

### Requirements for a novel method

Any new method needs to be both *reproducible* and *accurate* compared to the reference method. For example; a method that always quantifies the LVM to 150 g is very reproducible but inaccurate and not able to detect differences. A method with solely high accuracy is not useful if reproducibility is poor when serial measurements are needed. Accuracy was evaluated according to agreement with CMR^16,18^. Reproducibility was evaluated according to test–retest variability and according to intra/inter-examiner variability. Feasibility was evaluated in the study population and among all-comers. The impact of image quality on accuracy and reproducibility was evaluated. Sensitivity and specificity for detecting hypertrophy was calculated. As envisaged, methods based on 2DE/3DE demonstrate better accuracy than 1DE. The novel method seems more accurate than other 1DE/2DE-methods, has high reproducibility, is equally affected by image quality compared to the other 2D/3D-methods recommended by guidelines^6^, demonstrates high sensitivity and specificity for hypertrophy (Table 6) and, moreover, performs best regardless of LV geometry (Fig. 5). BA plots (Fig. 4B) also reveal equal distribution and limited proportional bias, based on the regression line. An informative overview of the various methods are presented in Table 7.

**Table 7:** Advantages and disadvantages of the different methods to quantify left ventricular mass.

	3DE	Novel	BP	TE	A-L	DEV
Number of images required	1	3	2	2	2	1
Image acquisition	↓	↔	↔	↔	↔	↑
Image analysis	↓	↔	↓	↔	↔	↑
Analysis time	↓↓	↔	↓	↔	↔	↑↑
Feasibility	↔	↔	↓	↔	↔	↑
Accuracy	↑	↑	↔	↔	↔	↓
Accuracy independent of left ventricular geometry	↓	↑	↓	↓	↔	↓
Test–retest variability	↑	↑	↓	↑	↑	↓
Intra/inter-examiner variability	↓	↑	↔	↑	↑	↓↓
Accuracy/reproducibility affected by image quality	↓	↔	↔	↔	↓	↔
Sensitivity (good at ruling out hypertrophy)	↔	↑↑	↑	↓	↑↑	↓↓
Specificity (good at ruling in hypertrophy)	↑	↑↑	↔	↑	↑↑	↑
Reliable outcome validation	↔	↓	↓	↓	↓	↑

↔ Standard, ↑↑ Advantageous, ↓↓ Disadvantageous.

*3DE* three-dimensional echocardiography, *BP* biplane model (both endo- and epicardial delineation), *TE* truncated ellipsoid, *A-L* area-length, *DEV* cube formula Devereux correction.

### The novel method versus conventional “linear” one-dimensional methods

**Figure 6:**
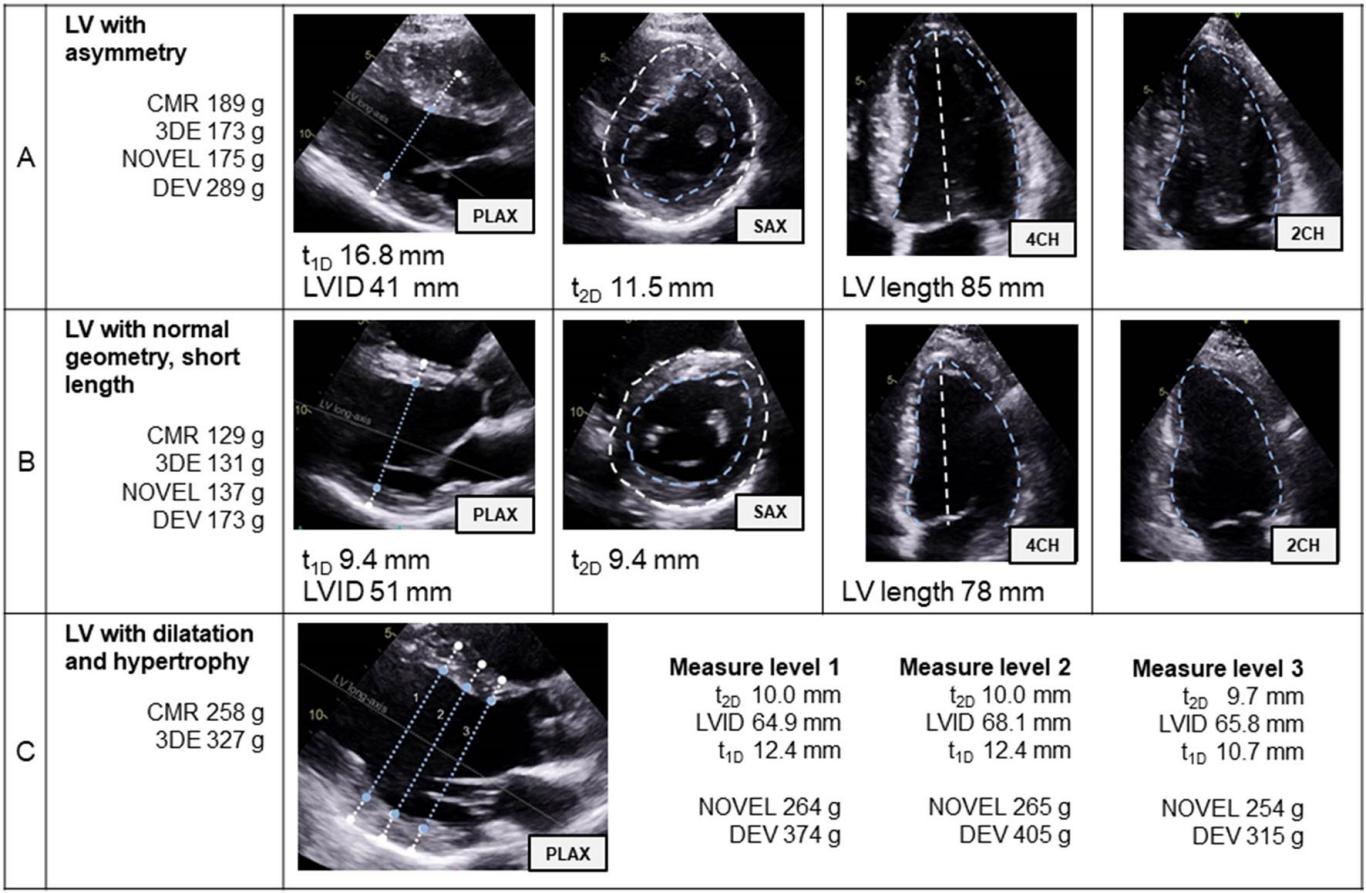
Examples of miscalculation by linear measurements. (**A**) Hypertrophic cardiomyopathy, asymmetry. (**B**) Normal geometry, short LV length. (**C**) Aortic regurgitation, dilatation and hypertrophy. *LV* left ventricle, *CMR* cardiac magnetic resonance, *3DE* three-dimensional echocardiography, *DEV* cube, Devereux correction, *PLAX* parasternal long-axis, *1D* one-dimensional, *t*_*1D*_ mean wall thickness PLAX, *LVID* left ventricular internal diameter, *SAX* short-axis, *2D* two-dimensional, *t*_*2D*_ mean wall thickness SAX, *4CH* four-chamber view, *2CH* two-chamber view.

It is unsurprising that the cube formula^11^ has been the most common method for LVM-quantification since the 1970’s, as this method is simple, feasible and useful in large population studies^1–3^. However, its simplicity makes it susceptible to measurement errors that make it less suitable for individual and serial measurements. For instance, recording a LVID of 45 mm with a small wall thickness measurement error of 11 mm instead of 10 mm yields 14% increase in LVM. Whereas wall thickness recordings using the novel method are derived from the whole circumferential area, and less sensitive to minor measurement errors. This vulnerability of methods deploying linear measurements to small differences that impact LVM measurement is reflected in the increased test–retest- and intra-/inter-examiner variations for DEV compared to the novel method (Table 3). High variations indicate decreased reproducibility and less ability to identify small yet significant real differences. Compared to the conventional method using DEV, our novel method presents lower variations and is thereby much more suited for monitoring serial measurements and comparing measurements by different examiners. Figure 6 demonstrates three patients with excellent image quality where methods deploying linear measurements fail to accurately quantify LVM: Example A has hypertrophic cardiomyopathy (HCM) and asymmetry, focal septal hypertrophy results in overestimation of *t* and consequently overestimation of LVM by 100 g. Whilst no echocardiographic method is ideal in focal/asymmetric HCM, 2DE/3DE correlate substantially better with CMR compared to 1DE. Example B has normal geometry with normal LVM and EDV_ENDO_. However, the LV is short, only 78 mm, predisposing to overestimation of the LV length and consequently LVM by 44 g. Other methods overcome this pitfall and correlate better with CMR. Example C has severe aortic regurgitation, the LV is both dilated and hypertrophied. Small measurement errors are particularly magnified amongst patients with large LVs, resulting in both overestimation and large variations of 315–405 g despite very small, almost visually undetectable measured differences. This is also illustrated in Fig. 5 where this patient group “dilatation and hypertrophy” has large SDs, particularly amongst the method utilising linear measurements. Variations in LV geometry and size are common in cardiac disease, a cohort that particularly requires correct LVM-quantification, warranting exploration of improved methodologies.

### The novel method versus other two-dimensional methods

Like A-L and TE, the novel method is based on adding the mean wall thickness from a single SAX-recording. What differs the novel method from these other two conventional 2D-methods is the biplane delineations of the endocardium in the apical 4CH- and 2CH-view, which preserves the LV geometric variations. Compared to the other 2D-methods, the novel method demonstrates similar feasibility of 95% in the study population (Table 3) and 81% among all-comers, slightly better reproducibility of 9% vs 10–11%, better agreement to CMR (Table 4) and overall better sensitivity, specificity, PPV and NPV (Table 6). Traditionally, the biplane model is applied for LV volumes and function and is better at correcting shape distortions compared to 1D-volume by Teichholtz or 2D-volume by A-L^6^. The biplane model has also been used for LVM-quantification; previous studies^7,22–27^ only report endo- and epicardial border delineation (Fig. 3C), not quantification (Fig. 3B). It is our experience that, the epicardium is more difficult to delineate than the endocardium. We envisaged advantages in measuring the myocardial thickness in another representative view and adding it to the EDV_ENDO_ to build up the EDV_EPI_. Compared to the BP-method, the novel method showed better feasibility, reproducibility and agreement to CMR. Several factors contribute to this observation: (1) reduced lateral resolution along the LV-walls impairs epicardial delineation in the apical views, (2) epicardial dropout, (3) echogenic pericardium, 4) small rotational errors causing the right ventricular wall to interfere with the epicardium of the inferior LV wall in the 2CH-view.

### The novel method versus 3D echocardiography

The overall superiority of the novel method compared to 3DE is better reproducibility, better sensitivity and specificity for hypertrophy (Table 3) and better accuracy across all four defined LV geometries (Fig. 5). The novel methods require standard 2D images whereas it is our experience that 3D image acquisition is more troublesome. Improved 2D image quality with high spatial and temporal resolution may contribute to these findings. Furthermore, post-processing analysis of 3D images requires specific training and is more time-consuming; 3DE 142 s and Novel 79 s (Table S8, Supplementary data). On the contrary, the favourable characteristics of 3DE compared to the novel methods is the lack of geometrical assumption regarding LV shape and distribution of LV hypertrophy. Shape distortions beyond the 4CH/2CH-views are not accounted for in the novel method and, inherent to other 2DE-methods, inaccurate apical images and LV-foreshortening may underestimate the EDV_ENDO_ and LVM. The 3DE-agreement to CMR in our study is consistent with recent studies^8^, however, we observed underestimation of LVM by 30 g among subjects with hypertrophy (Fig. 5), mostly HCM. Only 62% of the patients with geometry profiles of “hypertrophy” were correctly classified as being hypertrophic by 3DE (Table S9 Supplementary data) compared to 92% for the novel method. This is similar to Chang et al.^28^, who also report underestimation of 20 g and similar LOA in HCM, probably secondary to interpolation of small segments of the epicardium in the apex. Because of their larger LVs, the group with both hypertrophy and dilatation should also be prone to potential errors caused by interpolation of the epicardium in the apex, but we didn’t observe the same pattern in this group. A plausible explanation for our findings may be slight overestimation of LVM by CMR in subjects with HCM and small/normal EDV. It may be hard to distinguish between trabeculae and LV cavity; delineation is easier among hypertrophied patients with increased EDV. Recognised difficulties in LVM-quantification amongst HCM are illustrated by relatively large LOAs’ when comparing 3DE to CMR^29,30^.

### Estimation of mean wall thickness

We recommend acquiring *t* using 2DE delineations in SAX (Fig. 1, right panel), where it is easier to ensure centred/aligned measurements. Initially we compared both linear measurements in PLAX as 1DE and delineations in SAX as 2DE at three LV levels; mitral, chordae, mid-papillary (Fig. S1 Supplementary data). 2DE at chordae level performed slightly better (Table S3 Supplementary data). Current guidelines encourage 1DE-measurements at the mitral valve leaflet tip in PLAX and 2DE-delineations at the mid-papillary level in SAX^6^. However, results from Chetrit et al^31^, Guzzetti et al^32^ and our findings suggest that the measurement level corresponding to the mitral valve leaflet tip provides inaccurate quantification of LVM and that the preferred level is located more towards the mid-ventricular level. Similar to A-L, TE, and also DEV; the novel method is based on wall thickness estimation from a single imaging plane. Patients with distal wall thinning as in LV aneurysms, or basal septal hypertrophy are at risk of LVM-overestimation^32^, conversely patients with focal hypertrophy are at risk of LVM-underestimation. Measurements at several cross-sectional levels; at both base and apex may be considered with manifestly asymmetric geometry, although this may affect the feasibility and simplicity of the novel method. We did not include any patients with LV aneurysm. Because of deviating LV geometry in this patient group, the novel method and 3DE would hypothetically be more suited because of partial and full preservation of the LV shape. Furthermore, serial measurements with the novel method should not be particularly affected among patients with LV aneurysm, since the mass of fibrotic tissue does not have the ability to hypertrophy and remain constant over time.

### Future aspects regarding implementation of the novel method

We aimed to improve and facilitate echocardiographic LVM-quantification by developing a method that is simple, reproducible, accurate and reliable for monitoring individuals using serial measurements, without impairing workflow. This novel method does not require specific training and has substantially less post-processing analysis time than 3DE (Table S8, Supplementary data). Time-efficacy may be even further improved by applying simultaneous bi-plane acquisition^33^, which most vendors provide already. Once integrated with the vendor specific echocardiographic analysis software, our novel method will provide an automated LVM, comparable to LVM by 3DE or CMR and with high reproducibility. Thus, it provides a useful supplement to the modern echocardiographic report.

## Limitations

Our study population comprised mostly men (67%), young subjects (44 ± 14 years) with low BMI (25.5 ± 4.2 kg/m^2^), which may limit the applicability to other populations. We also recognize that developing and testing the model on the same population, may have biased the results and a validation cohort may have increased the strength of this study. Although all measurements were performed blinded, we were not blinded to the purpose of the study, which may have affected the results. We did not investigate potential vendor differences between 3DE and CMR. Further studies with various vendors or machines, contrast echocardiography, with/without contrast and with greater subject heterogeneity (including ages, obesity, LV shapes, hypertrophy and cardiac disorders) would provide corroboration, and widen interpretation and applicability of the findings. The novel method is not yet validated according to normal LVM-range or outcome. We recognise our report may pose challenges to interpreting established data that relied on less reproducible methodologies, although this is not unique to our observations/findings; future data is usually developed after adopting newer technologies and methods following a period of transition. For the subgroup without cardiac disease and without cardiovascular risk factors we found that LVM by the novel method ranged from 46 to 95 g/m^2^. We await validated normal LVM-ranges for both the 2DE/3DE-methods and hopefully, in time also for the novel method. We also recognize that there may be uncertainty regarding conventional geometrical classification according to relative wall thickness (RWT) and LVM index. Many clinical guidelines today are based upon linear measurements in PLAX. However, these measurements may also be ascertained by converting area to diameter using SAX-delineations. Theoretically, this may be a more accurate way of achieving RWT, since all segments of the LV are represented, not only the anterior–posterior segments.

## Conclusion

Our novel method for LVM-quantification is simple, has considerable higher reproducibility and accuracy compared to conventional “linear” 1DE, and similar accuracy as 3DE. It can easily be integrated into any vendor specific echocardiographic software, and as the biplane model forms part of the standard echocardiographic protocol it does not require specific training and provides a supplement to the modern echocardiographic report.

## Supplementary Information

Supplementary Information.

## Data Availability

The data that supports the findings of the current study are available from the corresponding author upon reasonable request.
