# Supplementary material for: A new method to quantify left ventricular mass by 2D echocardiography
Source: Sci Rep. 2022 Jun 15;12:9980. doi: 10.1038/s41598-022-13677-1 (PMC9200734; doi:10.1038/s41598-022-13677-1)
Supplement: Supplementary file 1 — Supplementary Information. [file 41598_2022_13677_MOESM1_ESM.docx]

**SUPPLEMENTARY DATA**

**A new method to quantify left ventricular mass by 2D echocardiography**

Charlotte Burup Kristensen, MD

The Department of Cardiology, Copenhagen University Hospital Rigshospitalet, Denmark

E-mail: charlotte.burup.kristensen@regionh.dk

**Supplementary Methods**

To find the version with the best agreement according to the reference method by cardiac magnetic resonance (CMR), the following were considered:

1) Mean wall thickness (*t*) by one-dimensional (1D) or two-dimensional (2D) methods

2) Measurement level for *t*

3) The height of the apical cap

4) A factor to adjust for inter-modality differences of end-diastolic volume (EDV)

These considerations provided us 12 different potential models for left ventricular mass (LVM) quantification. The best agreement was found in the version highlighted in the paper **(Figure S3)**.

**Acquisition of mean wall thickness (*t*) from one-dimensional echocardiography (1DE) or two-dimensional echocardiography (2DE)**

From the parasternal long-axis view (PLAX) **(Figure S1A)**: *IVS* interventricular septum, *LVPW* left ventricular posterior wall

$$t_{1D}=\frac{IVS+LVPW}{2}$$

From the short-axis view (SAX) **(Figure S1B)**: *A_1_* the traced area defined by the epicardial interface in the short-axis view (SAX), *A_2_* is the traced area defined by the endocardial interface in SAX.

$$t_{2D}=\left( \sqrt{\frac{A_{1}}{\pi}} \right)-\left( \sqrt{\frac{A_{2}}{\pi}} \right)$$

**Measurement level**

We considered three different measurement levels; the mitral valve, chordae level and the mid-papillary level in both PLAX for 1DE and SAX for 2DE **(Figure S1A-B)**. The measurement levels were applied to all the methods we compared where measurement level must be considered; two initial versions of our novel method (NOVEL_1D_ and NOVEL_2D_), the cube formula with Devereux correction (DEV), the Truncated Ellipsoid (TE) the Area-Length (A-L)-method.

**The height of the apical cap**

The apical cap was quantified using the geometrical assumption of a prolate half ellipsoid. We applied the epicardial radii of the most apical discs (disc number 30) of the four-chamber (4CH)- and two-chamber (2CH)-view and the height of 1.0*t* or 0.5*t* **(Figure S2)**. We hypothesized that the apical myocardium might be slightly thinner than the rest of the left ventricle (LV). Accordingly, we tested two versions of the apical cap, one applying the height of 1.0*t* (apical myocardium same thickness as the rest of the LV) and one applying the height of 0.5*t* (apical myocardium slightly thinner). The two versions of the apical cap were calculated as follows.

Version 1: (height = 1.0*t*)

$$V_{apical cap}=\frac{2}{3}\pi\left( t+\sqrt{kb_{30}b_{30}} \right)\left( t+\sqrt{kc_{30}c_{30}} \right)t$$

Version 2: (height = 0.5*t*)

$$V_{apical cap}=\frac{2}{3}\pi\left( t+\sqrt{kb_{30}b_{30}} \right)\left( t+\sqrt{kc_{30}c_{30}} \right)\frac{t}{2}$$

**The factor*, k***

End-diastolic volume (EDV) by 1DE, 2DE, 3DE and CMR divided by geometry is presented in **Table S1**. In line with previous studies, we report underestimation of EDV by both 2DE[1] and 3DE[2, 3] compared to CMR. Because of inter-modality differences of EDV by the biplane model (BP) and CMR, we applied a factor, *k*. The factor was calculated as follows:

$$k=\frac{1}{{{EDV}_{echo}}/{{EDV}_{cmr}}}$$

The factor, *k* was calculated to be 1.33±0.16 (mean±standard deviation). To validate *k* we compared EDV from 2DE by the biplane model of discs and CMR nine studies between year 2007 and 2020 with 622 subjects[4–12]. We found that the weighted total volumes from the studies, *k* was estimated to 1.32 **(Table S2)**. We await further studies to identify the optimal *k* to fit our model. Also, as a result of future improvements in imaging we envisage *k* may change accordingly. It is important to highlight that the factor *k* was only added to the EDV and not directly to the LVM or *t*. We shall try to explain the application of *k* in a stepwise manner; *first* the conventional EDV was quantified by the biplane model of discs, *then* the conventional EDV was adjusted so that the EDV corresponds to the EDV of the reference method using CMR and *thereafter* the *t* was added to the corrected EDV. Naturally, the *t* added to the corrected EDV will yield a larger myocardial volume and LVM compared to *t* added to the uncorrected EDV. It is also important to understand that the actor *k* in principle only adjusts the numerical difference (bias) between echocardiography and cardiac magnetic resonance but not the actual performance of the method. Limits of agreement to the reference method and reproducibility remains unchanged.

**The model with best agreement**

**Table S3** demonstrates the 12 versions of the novel method according to various levels the measurements are made within the LV and the height of the apical cap. The best agreement was found when combining *t* from 2DE at the chordae-level and height of the apical cap of 0.5*t*.

**Implementation of the novel method**

To apply the novel method, the following data are needed from the echocardiographic software **(Figure S3)**:

- the radius (*b*) from 30 unique discs in the 4CH-view

- the radius (*c*) from 30 unique discs in the 2CH-view

- the endocardial length of the LV in the 4CH-view (L_4CH_)

- the endocardial length of the LV in the 2CH-view (L_2CH_)

*t* was provided from tracings in SAX, as described in the main paper, but may also be derived from linear measurements in PLAX.

***Observe that number of discs may variate between vendors.***

*k* was applied to each unique endocardial disc sub-volume. Each unique disc is geometrically considered a short cylinder. The sub-volume of each unique disc (n=30 for each view) was calculated:

$$V_{4CH:disc}=k\pi b^{2}(\frac{L_{4CH}}{30})$$

$$V_{2CH:disc}=k\pi c^{2}(\frac{L_{2CH}}{30})$$

The LV volume defined by the endocardium (EDV_ENDO_) is the sum of 30 unique discs:

$${EDV}_{ENDO}=k\pi\sum_{i=1}^{30} b_{i}\times c_{i}\times\frac{\left( L_{4CH}+L_{2CH} \right)}{60}$$

The new volume of each disc was used to quantify the size of the new radius (*b_new_* or *c_new_*). We assumed that the inter-modal differences between CMR and 2DE mostly depends on differences in endocardial definition and not LV length, accordingly we allowed the LV to increase radially, but not in length.

$b_{new}=\sqrt{\frac{V_{4CH:disc}}{\pi\times\left( \frac{L_{4CH}}{30} \right)}}=\sqrt{\frac{k\pi b^{2}\left( \frac{L_{4CH}}{30} \right)}{\pi\times\left( \frac{L_{4CH}}{30} \right)}}=\sqrt{kb^{2}}$

$c_{new}=\sqrt{\frac{V_{2CH:disc}}{\pi\times\left( \frac{L_{2CH}}{30} \right)}}=\sqrt{\frac{k\pi c^{2}\left( \frac{L_{2CH}}{30} \right)}{\pi\times\left( \frac{L_{2CH}}{30} \right)}}=\sqrt{kc^{2}}$

The radius of the new disc defining the epicardial border was calculated by applying *t* to the new radius

$$b_{EPI}=t+\sqrt{kb^{2}}$$

$$c_{EPI}=t+\sqrt{kc^{2}}$$

The sub-volume of each unique disc defined by the epicardium was calculated:

$$\pi\left( t+\sqrt{kb^{2}} \right)\left( t+\sqrt{kc^{2}} \right)\left( \frac{L_{4CH}+L_{2CH}}{60} \right)$$

The sum of 30 unique discs/sub-volumes provided the total LV volume defined by the epicardium, without the apical cap:

$$\pi\sum_{i=1}^{30} \left( t+\sqrt{kb_{i}b_{i}} \right)\times\left( t+\sqrt{kc_{i}c_{i}} \right)\times\left( \frac{L_{4CH}+L_{2CH}}{60} \right)$$

Total LV volume defined by the epicardium (EDV_EPI_) was quantified by adding the apical cap:

$${EDV}_{EPI}=\left( \pi\sum_{i=1}^{30} \left( t+\sqrt{kb_{i}b_{i}} \right)\times\left( t+\sqrt{kc_{i}c_{i}} \right)\times\left( \frac{L_{4CH}+L_{2CH}}{60} \right) \right)+\left( \frac{2}{3}\pi\left( t+\sqrt{kb_{30}b_{30}} \right)\left( t+\sqrt{kc_{30}c_{30}} \right)\frac{t}{2} \right)$$

$$LVM=1.05\times\left( {EDV}_{EPI}-{EDV}_{ENDO} \right)$$

$$LVM=1.05\left\{ \left[ \left( \pi\sum_{i=1}^{30} \left( t+\sqrt{kb_{i}b_{i}} \right)\times\left( t+\sqrt{kc_{i}c_{i}} \right)\times\left( \frac{L_{4CH}+L_{2CH}}{60} \right) \right)+\left( \frac{2}{3}\pi\left( t+\sqrt{kb_{30}b_{30}} \right)\left( t+\sqrt{kc_{30}c_{30}} \right)\frac{t}{2} \right) \right]-\left[ k\pi\sum_{i=1}^{30} b_{i}\times c_{i}\times\frac{\left( L_{4CH}+L_{2CH} \right)}{60} \right] \right\}$$

**Differences in measurement levels for the conventional methods**

**Table S4** presents the feasibility of the echocardiographic methods and the agreement of LVM by various echocardiographic methods and CMR at three different levels of measurement within the LV. It is noticeable that the chordae level has the best agreement not only in the novel method, but also amongst the other methods where the level within the LV at which measurements are taken must be considered; the 1D method by DEV and the 2D methods by TE and A-L.

**Left ventricular geometrical classification**

**Table S5** presents the left ventricular geometrical classification according to age, gender, LVM index and cardiac condition for the whole population.

**Subgroup analysis of fifty-nine subjects with 100% feasible measurements**

To assure that differences in feasibility didn’t affect the results, we performed a subgroup analysis of the fifty-nine patients that had 100% feasible measures. **Table S6A** demonstrates the excluded patients. **Table S6B** demonstrates the geometrical classification of the subgroup. The results from this subgroup (n=59) **(Table S7)** indicate similar results as for the whole population.

**Time-efficacy of the novel method**

The novel method does not add any time to image acquisition, compared to the biplane method. To demonstrate the analysis time (the post-processing time) we performed a small subgroup analysis **(Table S8)** for the various methods. The analysis time for the novel method is substantially less than the 3DE-analysis time.

**Re-classification**

Correct classification and re-classification of hypertrophy was evaluated for each method. Classification and re-classification are presented in **Table S9**. The novel method classified 90% of the subjects correctly, which is better than the other methods. The novel method was able to re-classify 14 of 20 subjects with initial incorrect classification by 1DE.

**Supplementary Figures
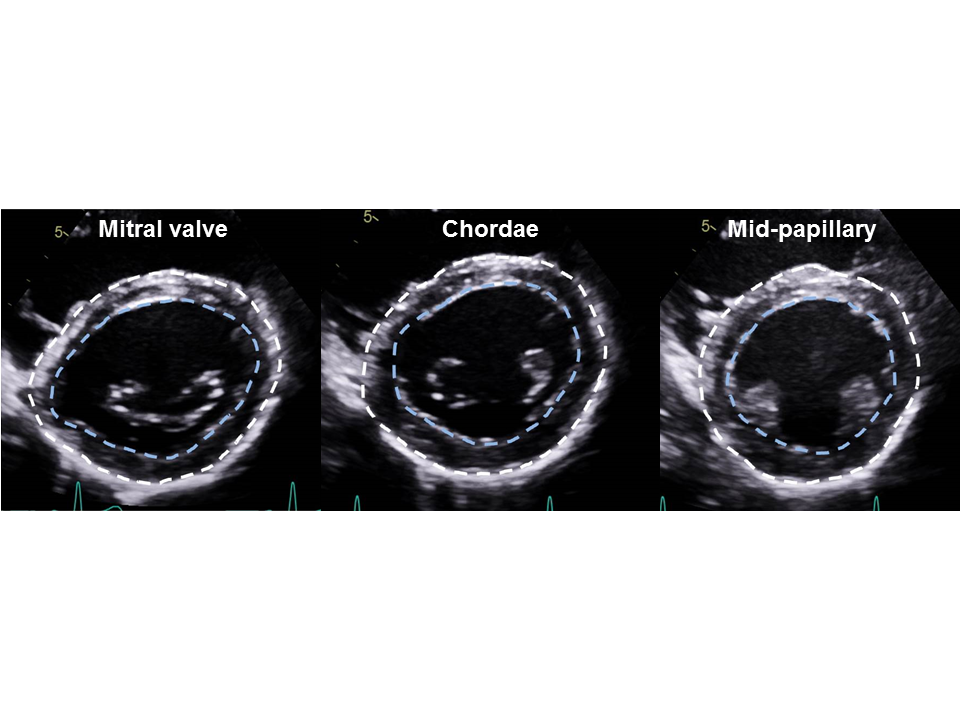
**
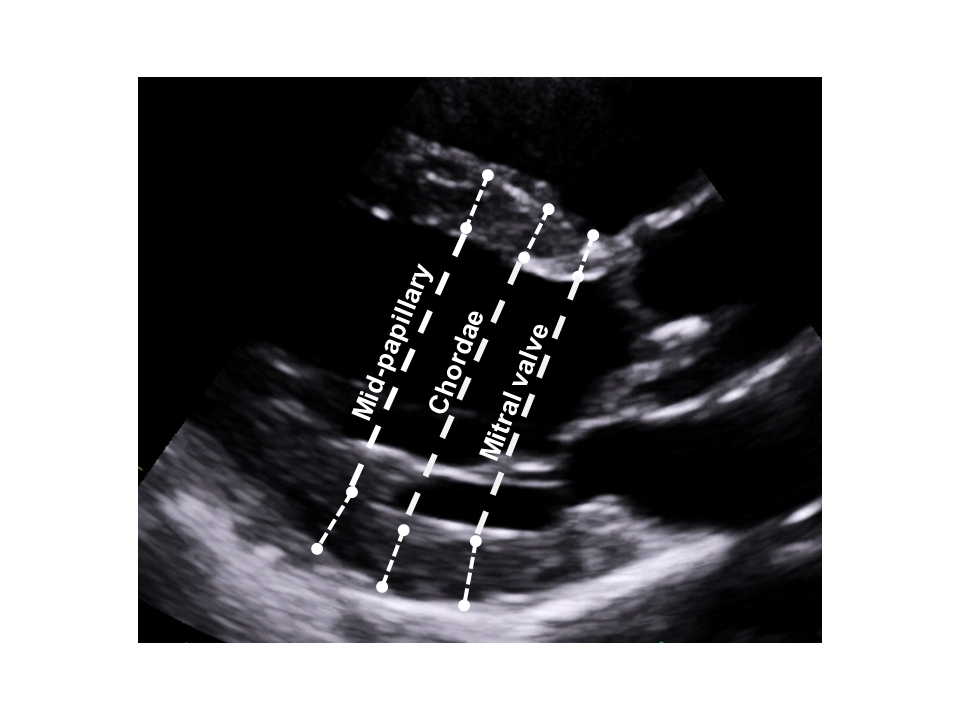


**Figure S1A:** Measurement levels in the parasternal long-axis view

Mitral valve-, Chordae- and Mid-papillary level

Mitral valve-, Chordae- and Mid-papillary level

**Figure S1B:** Measurement levels in the short-axis view


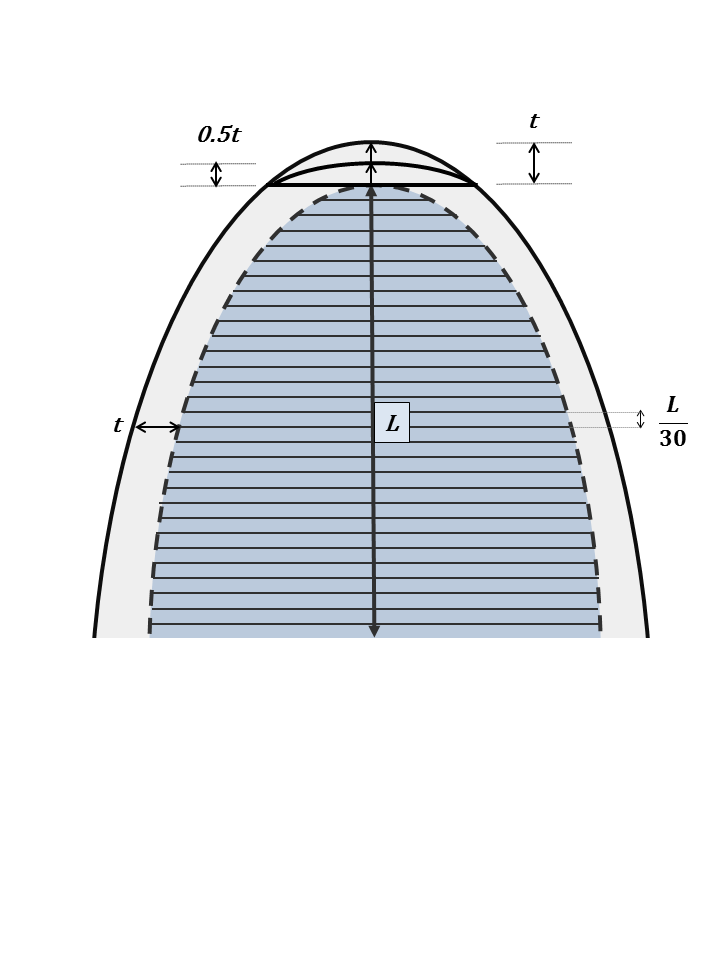


**Figure S2: Two versions of the apical cap.**

Blue area represents part of the endocardial tracing. The solid outer line represents the estimated epicardial border. Most upper part represents the apical cap in two versions; 1.0*t* and 0.5*t*

**
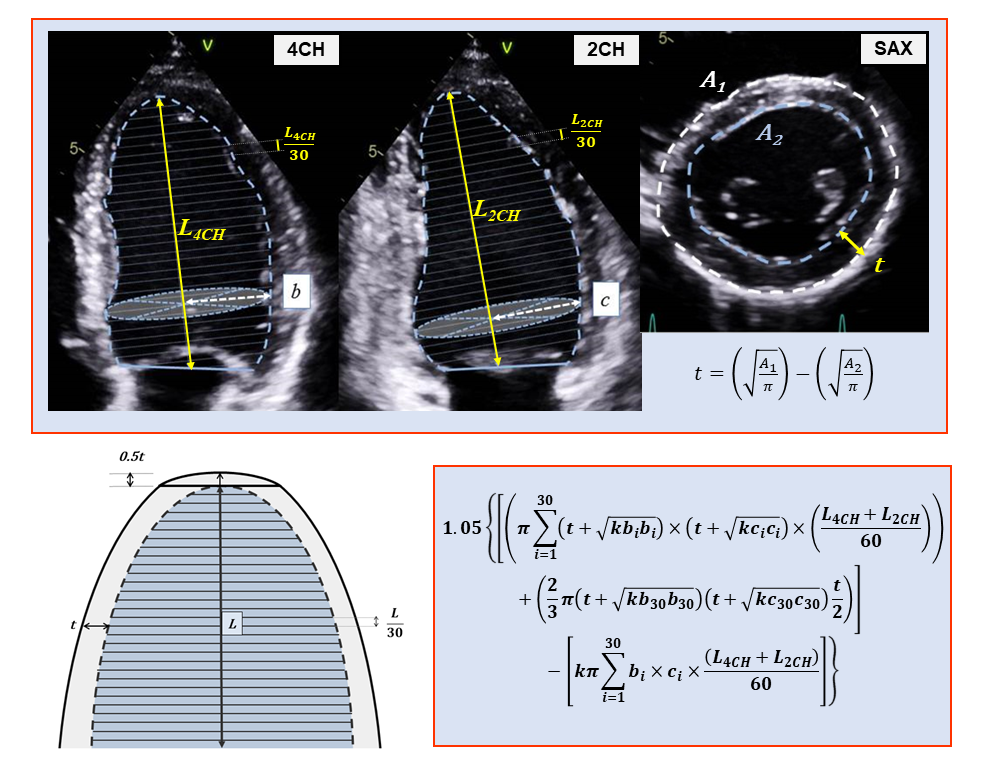
**

**Figure S3**: **The novel method**

Upper part: Required delineations and length measurements, radii (white arrows), endocardial discs (n=30 for each apical view) and height of the discs.

Lower left side: The novel method with the apical cap. Blue area delineated by black dotted line=endocardial traced volume. Black solid line=epicardial quantified volume.

Lower right side: formula

**4CH** four-chamber, **2CH** two-chamber, **SAX** short-axis, ***L*** left ventricular length, ***t*** mean wall thickness, ***b*** radius, unique disc in the four-chamber-view, ***c*** radius, unique disc in the two-chamber-view, ***A_1_*** outer delineation, ***A_2_*** inner delineation, ***k*** factor

**Supplementary Tables**

| **Table S1**  End-diastolic volumes at baseline; all patients and divided by the four defined left ventricular geometries | | | | | |
| --- | --- | --- | --- | --- | --- |
|  | **All** | **Normal** | **Dilatation** | **Hypertrophy** | **Dilatation and hypertrophy** |
| **End-diastolic volume (ml)** | mean±SD | mean±SD | mean±SD | mean±SD | mean±SD |
| CMR | 197±60 | 160±25 | 209±33 | 181±35 | 275±84 |
| 3DE | 147±51 | 114±25 | 155±29 | 148±38 | 205±73 |
| 2DE (Biplane) | 151±50 | 123±24 | 159±31 | 139±34 | 211±73 |
| 1DE (Teichholtz) | 131±43 | 110±22 | 141±38 | 109±31 | 177±55 |
| **SD** standard deviation, **CMR** cardiac magnetic resonance, **3DE** three-dimensional echocardiography, **2DE** two-dimensional echocardiography, **1DE** one-dimensional (linear) echocardiography | | | | | |

| **Table S2**  Estimation of *k* from other publications | | | | | | |
| --- | --- | --- | --- | --- | --- | --- |
| **Author** | **Publication year** | **Subjects** | **n** | **2D (ml)** | **CMR (ml)** | ***k*** |
| Jenkins et al[4] | 2007 | CAD | 30 | 111 | 168 | *1.51* |
| Bicudo et al[5] | 2008 | HCM | 20 | 134 | 135 | *1.01* |
| Jenkins et al[6] | 2009 | CAD | 50 | 125 | 207 | *1.66* |
| Marsan et al[7] | 2011 | LV aneurysm | 52 | 247 | 288 | *1.17* |
| Hoffmann et al[8] | 2014 | CAD and/or MI (mostly) | 63 | 107 | 175 | *1.64* |
| Haan et al[9] | 2014 | HF prior CRT/ICD | 152 | 213 | 283 | *1.33* |
| Jenner et al[10] | 2019 | MI | 32 | 122 | 181 | *1.48* |
| Losi et al[11] | 2019 | HCM | 61 | 85 | 137 | *1.61* |
| Schwaiger et al[12] | 2020 | MI | 162 | 135* | 151* | *1.12* |
| *Weighted volume (ml)* |  |  |  | *95,120* | *125,739* | *1.32* |
| *Volumes reported in this table are mean values from two separate subgroups in the study  **n** number of subjects, **2D** two-dimensional, ***k*** factor, **CAD** coronary artery disease, **MI** myocardial infarction, **HCM** hypertrophic cardiomyopathy, **LV** left ventricular, **HF** heart failure, **CRT** cardiac resynchronization therapy, **ICD** implantable cardioverter defibrillator | | | | | | |

| **Table S3**  Agreement between left ventricular mass from echocardiography and cardiac magnetic resonance | | | |
| --- | --- | --- | --- |
|  | **Bias±LOA** | ***CV (%)*** | ***r*** |
| **Height of apical cap = 0.5*t*** |  |  |  |
| 1DE PLAX: Mitral valve | -17±79‡ | *27* | *0.77*‡ |
| 1DE PLAX: Chordae | 0±71 | *22* | *0.85*‡ |
| 1DE PLAX: Mid-papillary | 3±77 | *23* | *0.85*‡ |
| 2DE SAX: Mitral valve | -5±57 | *18* | *0.90*‡ |
| 2DE SAX: Chordae | 2±50 | *15* | *0.92*‡ |
| 2DE SAX: Mid-papillary | 2±57 | *17* | *0.89*‡ |
| **Height of apical cap = 1.0*t*** |  |  |  |
| 1DE PLAX: Mitral valve | -15±80† | *27* | *0.77*‡ |
| 1DE PLAX: Chordae | 3±74 | *23* | *0.84*‡ |
| 1DE PLAX: Mid-papillary | 6±80 | *24* | *0.85*‡ |
| 2DE SAX: Mitral valve | -3±58 | *18* | *0.89*‡ |
| 2DE SAX: Chordae | 4±51 | *16* | *0.91*‡ |
| 2DE SAX: Mid-papillary | 4±59 | *18* | *0.89*‡ |
| ** p<0.05 † p<0.01 ‡ p<0.001*  The effect of measurement level and different heights of the apical cap for various versions (*n=12*) of the novel method to left ventricular mass quantification.  All values are agreement or correlation of echocardiographic left ventricular mass to cardiac magnetic resonance  **LOA** limits of agreement, **CV** coefficient of variation, ***t*** mean wall thickness, **1DE** one-dimensional (linear) echocardiography, **PLAX** parasternal long axis view, **r** Pearson’s correlation, **2DE** two-dimensional echocardiography, **SAX** short axis view | | | |

| **Table S4**  Echocardiographic methods and measurement levels | | | | |
| --- | --- | --- | --- | --- |
|  | **Feasibility** | **Agreement** |  |  |
| Method: level | *(%)* | Bias±LOA | *CV*  *(%)* |  |
| NOVEL_2D_: Mitral | 94 | -5±57 | *18* |  |
| NOVEL_2D_: Chordae | 95 | 2±50 | *15* |  |
| NOVEL_2D_: Mid-papillary | 96 | 2±57 | *17* |  |
| TE: Mitral | 94 | -21±61‡ | *20* |  |
| TE: Chordae | 95 | -2±54 | *17* |  |
| TE: Mid-papillary | 96 | -1±56 | *17* |  |
| A-L: Mitral | 94 | 10±66* | *20* |  |
| A-L: Chordae | 95 | 21±56‡ | *16* |  |
| A-L - Mid-papillary | 96 | 16±61‡ | *18* |  |
| NOVEL_1D_: Mitral | 84 | -17±79‡ | *26* |  |
| NOVEL_1D_: Chordae | 100 | 1±71 | *22* |  |
| NOVEL_1D_: Mid-papillary | 100 | 3±77 | *23* |  |
| DEV: Mitral | 84 | -18±77‡ | *26* |  |
| DEV: Chordae | 100 | 7±76 | *23* |  |
| DEV: Mid-papillary | 100 | 10±80* | *24* |  |
| * p<0.05 † p<0.01 ‡ p<0.001  Echocardiographic methods to estimate left ventricular mass where measurement level must be considered. Agreement between echocardiography and by cardiac magnetic resonance at baseline  **LOA** 95% limits of agreement, **CV** coefficient of variation, **NOVEL_2D_** novel method with mean wall thickness from two-dimensional tracings in the parasternal short-axis view, **TE** truncated ellipsoid, **A-L** area-length, **NOVEL_1D_** novel method with mean wall thickness from one-dimensional linear measurements in the parasternal long-axis view, **DEV** cube formula by Devereux | | | | |

| **Table S5**  Geometry-classification according to left ventricular mass and end-diastolic volume by cardiac magnetic resonance[13] | | | | | |
| --- | --- | --- | --- | --- | --- |
|  | All (n=85) | Normal (n=33) | Dilatation  (n=24) | Hypertrophy (n=13) | Dilatation and hypertrophy (n=15) |
| EDV_ENDO_ (ml), mean±SD | 197±60 | 160±25 | 209±33 | 181±35 | 275±84 |
| LVM (g), mean±SD | 165±62 | 126±32 | 148±30 | 241±76 | 215±44 |
| Control | 44 | 27 | 16 | 0 | 1 |
| HCM | 16 | 2 | 1 | 9 | 4 |
| DCM | 2 | 0 | 1 | 0 | 1 |
| ARVC | 1 | 0 | 0 | 1 | 0 |
| AS - moderate-severe | 6 | 2 | 0 | 2 | 2 |
| AR - moderate-severe | 6 | 0 | 0 | 0 | 6 |
| IHD | 3 | 2 | 1 | 0 | 0 |
| Others with cardiac disease | 7 | 0 | 5 | 1 | 1 |
| **LVM** left ventricular mass, **BSA** body surface area, **EDV_ENDO_** end-diastolic volume, endocardial, **SD** standard deviation, **HCM** hypertrophic cardiomyopathy, **DCM** dilated cardiomyopathy, **ARVC** arrythmogenic right ventricular cardiomyopathy, **AS** aortic valve stenosis, **AR** aortic valve regurgitation, **IHD** ischemic heart disease | | | | | |

| **Table S6A**  Excluded patient that did not have 100% feasible measures for all echocardiographic methods | | | | |
| --- | --- | --- | --- | --- |
|  | Normal  (-13) | Dilatation  (-5) | Hypertrophy  (-1) | Dilatation and hypertrophy  (-6) |
| Control | -13 | -5 | 0 | 0 |
| HCM | 0 | 0 | 0 | -1 |
| DCM | 0 | 0 | 0 | -1 |
| ARVC | 0 | 0 | 0 | 0 |
| AS - moderate-severe | 0 | 0 | 0 | 0 |
| AR - moderate-severe | 0 | 0 | 0 | -4 |
| IHD | 0 | 0 | 0 | 0 |
| All others with cardiac disease | 0 | 0 | -1 | 0 |
|  | | | | |

| **Table S6B**  Classification of the new subgroup (*n=59*) according to LVM and EDV by CMR | | | | |
| --- | --- | --- | --- | --- |
|  | Normal (n=20) | Dilatation (n=18) | Hypertrophy (n=12) | Dilatation and hypertrophy (n=9) |
| CMR EDV (ml), mean±SD | 160±24 | 218±31 | 179±35 | 249±67 |
| CMR LVM (ml), mean±SD | 128±32 | 157±24 | 243±79 | 211±47 |
| Control | 14 | 11 | 0 | 1 |
| HCM | 2 | 1 | 9 | 3 |
| DCM | 0 | 1 | 0 | 0 |
| ARVC | 0 | 0 | 1 | 0 |
| AS - moderate-severe | 2 | 0 | 2 | 2 |
| AR - moderate-severe | 0 | 0 | 0 | 2 |
| IHD | 2 | 1 | 0 | 0 |
| All others with cardiac disease | 0 | 4 | 0 | 1 |
| **HCM** hypertrophic cardiomyopathy, **DCM** dilated cardiomyopathy, **ARVC** arrythmogenic right ventricular cardiomyopathy, **AS** aortic valve stenosis, **AR** aortic valve regurgitation, **IHD** ischemic heart disease, **LVM** left ventricular mass, **EDV** end-diastolic volume, **CMR** cardiac magnetic resonance, **SD** standard deviation | | | | |

| **Table S7**  Agreement of left ventricular mass quantified by echocardiography and cardiac magnetic resonance (*n=59*) | | |
| --- | --- | --- |
|  | Bias±LOA | *CV(%)* |
| 3DE | -2±53 | *16* |
| NOVEL | -1±49 | *14* |
| BP | 6±60 | *17* |
| TE | -4±54 | *16* |
| A-L | 20±56 | *16* |
| DEV | 7±72 | *21* |
| **LOA** limits of agreement, **CV** coefficient of variation, **3DE** three-dimensional**, NOVEL** novel method with mean wall thickness from two-dimensional tracings in the parasternal short-axis view at the chordae level, **BP** Epi- and endocardial tracings by the biplane model of discs, **TE** truncated ellipsoid chordae level, **A-L** area-length chordae level, **DEV** cube formula by Devereux chordae level | | |

| Table S8  Time spent on post-processing analysis (n=10) | |
| --- | --- |
|  | **Analysis (s)** |
| 3DE | 142 ± 27 |
| NOVEL | 79 ± 8 |
| BP | 89 ± 11 |
| TE | 54 ± 3 |
| A-L | 47 ± 3 |
| DEV | 25 ± 4 |
| 3DE three-dimensional, NOVEL novel method with mean wall thickness from two-dimensional tracings in the parasternal short-axis view at the chordae level, BP Epi- and endocardial tracings by the biplane model of discs, TE truncated ellipsoid chordae level, A-L area-length chordae level, DEV cube formula by Devereux chordae level | |

| **Table S9**  Correct classification (hypertrophy/non-hypertrophy) and re-classification | | | | | | | |
| --- | --- | --- | --- | --- | --- | --- | --- |
|  | Correct classification | | | | | Re-classification | |
|  |  | *Non-hypertrophy* | | *Hypertrophy* | | *All* | |
|  | All n(%) | Normal (%) | Dilatation (%) | Hypertrophy (%) | Dilatation and hypertrophy (%) | Re-class. incorrect to correct, n | Re-class. correct to incorrect, n |
| 3DE | 68(82) | 91 | 83 | 62 | 80 | 11 | 6 |
| NOVEL | 73(90) | 94 | 96 | 92 | 73 | 14 | 3 |
| BP | 48(76) | 81 | 65 | 77 | 89 | 13 | 11 |
| TE | 67(83) | 97 | 100 | 67 | 40 | 6 | 1 |
| A-L | 71(88) | 90 | 87 | 92 | 80 | 14 | 5 |
| DEV | 65(76) | 94 | 100 | 39 | 33 | - | - |
| **3DE** three-dimensional echocardiography, **BP** biplane, **TE** truncated ellipsoid, **A-L** area-length, **DEV** cube, Devereux  **Re-class. incorrect to correct**, initially incorrectly classified by Devereux (n=20) and correctly re-classified  **Re-class. correct to incorrect**, initially correctly classified by Devereux (n=65) and incorrectly re-classified | | | | | | | |

**Supplement References**

1. Dorosz JL, Lezotte DC, Weitzenkamp DA, et al (2012) Performance of 3-dimensional echocardiography in measuring left ventricular volumes and ejection fraction: A systematic review and meta-analysis. J Am Coll Cardiol 59:1799–1808. https://doi.org/10.1016/j.jacc.2012.01.037

2. Shimada YJ, Shiota T (2012) Meta-analysis of accuracy of left ventricular mass measurement by three-dimensional echocardiography. Am J Cardiol 110:445–452. https://doi.org/10.1016/j.amjcard.2012.03.046

3. Kitano T, Nabeshima Y, Otsuji Y, et al (2019) Accuracy of Left Ventricular Volumes and Ejection Fraction Measurements by Contemporary Three-Dimensional Echocardiography with Semi- and Fully Automated Software: Systematic Review and Meta-Analysis of 1,881 Subjects. J Am Soc Echocardiogr 32:1105-1115.e5. https://doi.org/10.1016/j.echo.2019.04.417

4. Jenkins C, Leano R, Chan J, Marwick TH (2007) Reconstructed Versus Real-time 3-Dimensional Echocardiography: Comparison with Magnetic Resonance Imaging. J Am Soc Echocardiogr 20:862–868. https://doi.org/10.1016/j.echo.2006.12.010

5. Bicudo LS, Tsutsui JM, Shiozaki A, et al (2008) Value of real time three-dimensional echocardiography in patients with hypertrophic cardiomyopathy: Comparison with two-dimensional echocardiography and magnetic resonance imaging. Echocardiography 25:717–726. https://doi.org/10.1111/j.1540-8175.2008.00684.x

6. Jenkins C, Moir S, Chan J, et al (2009) Left ventricular volume measurement with echocardiography: A comparison of left ventricular opacification, three-dimensional echocardiography, or both with magnetic resonance imaging. Eur Heart J 30:98–106. https://doi.org/10.1093/eurheartj/ehn484

7. Marsan NA, Westenberg JJM, Roes SD, et al (2011) Three-dimensional echocardiography for the preoperative assessment of patients with left ventricular aneurysm. Ann Thorac Surg 91:113–121. https://doi.org/10.1016/j.athoracsur.2010.08.048

8. Hoffmann R, Barletta G, Von Bardeleben S, et al (2014) Analysis of left ventricular volumes and function: A multicenter comparison of cardiac magnetic resonance imaging, cine ventriculography, and unenhanced and contrast-enhanced two-dimensional and three-dimensional echocardiography. J Am Soc Echocardiogr 27:292–301. https://doi.org/10.1016/j.echo.2013.12.005

9. de Haan S, de Boer K, Commandeur J, et al (2014) Assessment of left ventricular ejection fraction in patients eligible for ICD therapy: Discrepancy between cardiac magnetic resonance imaging and 2D echocardiography. Netherlands Hear J 22:449–455. https://doi.org/10.1007/s12471-014-0594-0

10. Jenner J, Sörensson P, Pernow J, et al (2019) Contrast Enhancement and Image Quality Influence Two- and Three-dimensional Echocardiographic Determination of Left Ventricular Volumes: Comparison With Magnetic Resonance Imaging. Clin Med Insights Cardiol 13:. https://doi.org/10.1177/1179546819831980

11. Losi MA, Imbriaco M, Canciello G, et al (2020) Left Ventricular Mass in Hypertrophic Cardiomyopathy Assessed by 2D-Echocardiography: Validation with Magnetic Resonance Imaging. J Cardiovasc Transl Res 13:238–244. https://doi.org/10.1007/s12265-019-09911-3

12. Schwaiger JP, Reinstadler SJ, Tiller C, et al (2020) Baseline LV ejection fraction by cardiac magnetic resonance and 2D echocardiography after ST-elevation myocardial infarction – influence of infarct location and prognostic impact. Eur Radiol 30:663–671. https://doi.org/10.1007/s00330-019-06316-3

13. Kawel-Boehm N, Maceira A, Valsangiacomo-Buechel ER, et al (2015) Normal values for cardiovascular magnetic resonance in adults and children. J. Cardiovasc. Magn. Reson. 17
